# Structure and dynamics of liposomes designed for drug delivery: coarse-grained molecular dynamics simulations to reveal the role of lipopolymer incorporation

**DOI:** 10.1039/c9ra08632c

**Published:** 2020-01-22

**Authors:** Mohammed Lemaalem, Nourddine Hadrioui, Abdelali Derouiche, Hamid Ridouane

**Affiliations:** Laboratoire de Physique des Polymères et Phénomènes Critiques Sciences Faculty Ben M'Sik, Hassan II University P. O. Box 7955 Casablanca Morocco mohammedlemaalem@gmail.com

## Abstract

In this work, coarse-grained molecular dynamics simulations are carried out in NPTH and NVTE statistical ensembles in order to study the structure and dynamics properties of liposomes coated with polyethylene glycol (PEG). The considered liposomes are made by membrane bilayer DPPC with DPPC-PEG incorporated lipopolymers, in an aqueous environment. We have described the two essential PEG conformation regimes, mushroom and brush, and their properties which depend on the grafting density. The effects of grafting density on the structure and dynamics of the membrane were also studied. Our simulations were then discussed by comparing with the available experimental results and by referring to the suitable theoretical models. The results from the NPTH simulations agree with the experimental data of X-ray diffraction and with scale and mean-field theories in terms of thickness of the PEG layer and thickness of the DPPC bilayer membrane. The results from NVTE simulations are found in good agreement with the experimental results from studying the diffusion of the DPPC bilayer membrane and the PEG. The analysis of the mean square displacement revealed that the dynamics of the membranes in the plane show a subdiffusion due to the cage effect and that the grafted PEG dynamics is better described by the Rouse diffusion-mode. Thus, from a macroscopic viewpoint, the incorporation of DPPC-PEG plays an important role in the protection and lubrication of the liposome.

## Abstract 2

In this work, coarse-grained molecular dynamics simulations are carried out in NPTH and NVTE statistical ensembles in order to study the structure and dynamics properties of liposomes coated with polyethylene glycol (PEG).

## Graphical abstract

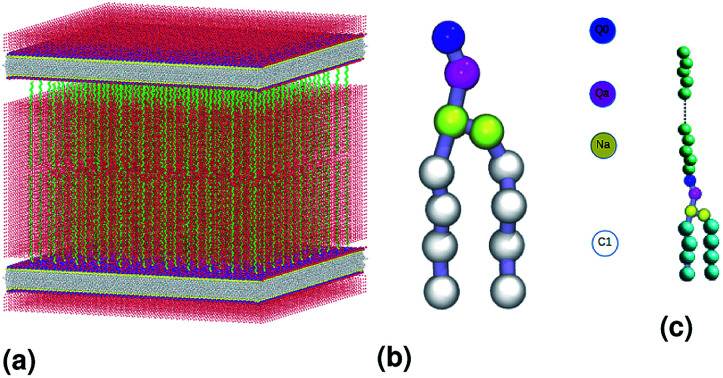

## 1 Introduction

Supported lipid bilayer membranes have a growing interest in studying the structure and function of membrane proteins and receptors.^1–3^ In order to increase the spacing between the hydrophilic support and the bilayers, a random polymer is introduced into this space in a controlled manner. This is supposed to increase the dynamic flexibility of the supported bilayer and allow the incorporation of large membrane proteins. This new concept has been used to incorporate integral membrane protein functions.^4,5^ Polymer-grafted lipid bilayers have several roles in drug delivery,^5,6^ such as the stabilization of liposomes,^6^ synthesis of supported bilayers for biomaterial applications,^7^ modification of the surface of implanted medical devices and protection of cytotoxic drugs.^8^ It has also been found that graft lipopolymers stabilize liposomes sterically and provide protection against immune system attacks and antibiotic resistance.^9,10^ Polymers play an important role in the synthesis of supported membranes where the bilayer rests on a polymer cushion tethered to a solid substrate. These polymer-supported membranes have shown remarkable stability in air and have the potential to be used for *in vitro* biosensor applications.^11,12^ A polymer commonly used in grafting studies is polyethylene glycol (PEG), which is hydrophilic and biocompatible. Cell adhesion is a phenomenon that is essential for most physiological cellular phenomena such as, survival, differentiation, migration, and activation.^13–15^ It is also essential for pathological situations such as the formation of cancerous metastases, tissue invasion by a pathogenic agent, inflammation, and reaction of the organism with respect to biomaterials.^16–18^ Liposomes are made out of the same material as a cell membrane, so they are supposed to the adhesion phenomena. The incorporation of lipopolymers can be an efficient solution to prevent the adhesion between liposome–liposome and between liposome–human cells. The PEG coating acts as a shield against the hydrophobic properties of the target organ cells that tend to stick the liposomes on their membranes. Everything happens as if it added some kind of lubricant. The quantitative study of the physical properties of the polymer serves to find the most effective parameters for acting on the target cells. Thus, PEG covered liposomes would be an alternative to the use of nanoparticles to deliver treatments of different pathologies, especially anticancer treatments.

For an experimental viewpoint, several microscopy techniques have been used to examine supported lipid bilayers, such as quartz crystal microbalance (QCM-D),^19^ surface plasmon resonance,^20^ neutron reflectivity,^21^ microscopy to atomic force,^22,23^ spectroscopy and ellipsometry,^24^ and the fluorescence interference contrast microscopy (FLIC).^25^ Quartz crystal microbalance (QCMD) is a microscopy technique that tracks adsorption, fusion, and rupture of vesicles in different types of surfaces in real time. The neutron reflectivity with the labeled deuterium components is a relatively well-established technique for measuring different distances of the diffusion density layer in the supported membranes. However, this technique requires relatively large amounts of hardware, expensive equipment, and data analysis depending on the model. Neutron reflectivity measurements also have a poor lateral resolution. Surface plasmon resonance and quartz microbalance techniques simply detect the refractive index and surface mass changes and are therefore insufficient to solve the structural details of the supported bilayers. The fluorescence interference contrast microscopy (FLIC) was developed to measure cell/substrate distances but don't give information about the nanostructure, dynamics and thermodynamic information. Volker Kiessling and Lukas K. Tamm measured the distance of polyethylene glycol between the supported lipid bilayers and the surface of the oxidized silicon chips by fluorescence interference contrast microscopy (FLIC). They found a distance ranging from 1.7 to 3.9 nm, depending on the molecular weight of grafted polymers,^25^ Lambacher and Fromherz measured the distance of poly (2-methyl-2-oxazoline) between the substrate and bilayers supported by fluorescence contrast microscopy (FLIC). They found that, for a polymer fraction of 0.5, the increase in the degree of polymerization from *n*_p_ = 33 to *n*_p_ = 104 resulted in an increase in the distance from *d* = 2.3 ± 0.7 nm to *d* = 4.8 ± 0.6 nm.^26^ Note that there are several prerequisites, especially for making and controlling adhesive surfaces defined on a subnanometric scale, and for understanding the Brownian motion of a particle in the vicinity of a wall. To this end, we propose to associate the molecular dynamics simulation (MD) method with the traditional tools of cell biology, to measure and exploit the phenomenon of liposomes adhesion, this method is promising in biophysics to explore the details of the most important physical properties with a good agreement with the available experimental techniques. MD also opens a wide range of studies by modifying experimental parameters and conditions, particularly for complex biological systems. For a dynamic point of view, the subdiffusion behavior, predicted by several theoretical approaches and by experimental methods designed to probe motion at picosecond timescales, can be evaluated using all-atom MD simulation and as well as by coarse-grained MD simulations. For the time being there is no simple and satisfactory theoretical model to describe the subdiffusion phenomena caused by the cage effect, as the later can depend on several physicochemical parameters. For this consideration, MD method, which is based on empirical interaction potentials, is a preferred tool for evaluating the anomalous and slow dynamical behavior, as it separately determines the motion of each particle in the simulated system. The document is organized as follows: in Section 2, we present the details of the performed simulations. In Section 3, the distance between two membranes with PEG-grafted polymers is estimated by using molecular dynamics simulations in the NPHT statistical ensemble and is compared with that obtained by experiment, then a study of the dynamic and structural properties of the DPPC-PEG lipopolymers and the membrane lipids is carried out in the NVTE statistical ensemble.

## 2 System model and simulation method

### 2.1 Principle of molecular dynamics

Coarse-grained Molecular Dynamics (CG-MD) simulation is a dynamical simulation technique, in which the equations of motion for a system of particles are numerically integrated over time to obtain particle trajectories in phase space. In contrast to all-atom MD simulation, which usually describes microscopic particles (atoms, or at most small united atom clusters), CG-MD aims can describe particle systems at the coarse-grained mesoscopic level. Accordingly, the point particles in a model describe center-of-mass positions of atoms, clusters, molecules or subsections of polymeric molecules. The principle of CG-MD simulation is particularly simple and consists of generating the trajectories of a finite ensemble of CG beads, by integrating numerically the classical equations of motion *F*(*x*_*i*_) = −∇*U*(*x*_*i*_) = *M*_*i*_*V̇*_*i*_(*t*). The potential energy function *U*(*x*_*i*_) of the bead (*i*) is a function of the particle coordinates *x*_*i*_, it is the applied interaction by the other beads in the chosen force field, and *M*_*i*_ is the mass of the CG bead (*i*). Each particle trajectory is resolved separately. Thus, the determined trajectories are used to evaluate the static and dynamic properties, passing by some statistical physics equations. It should be noted that, the temporal averages, at very large-times, coincide with the statistical-averages, for ergodic systems. Indeed, the evolution of the positions and velocities of the N beads is obtained by solving a system of motion equations. Such an equation can be integrated numerically using the Verlet algorithm, to give the trajectories of the beads in phase space in each time-step.1
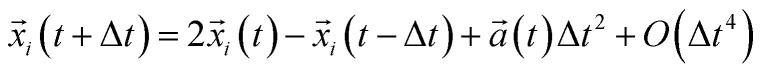


At each time-step, as well as for the particle positions, any physical quantity takes instantaneous value. When the system is at thermodynamic equilibrium, the static properties becomes stationary, and its average value can be determined within a time interval. Thus, MD makes the study of the behavior of the considered system possible, on average, by calculating its temporal evolution numerically and averaging the quantity of interest over a long enough time. In the framework of MD simulation, it is necessary to compute the forces on all the beads and to update all the positions. The principle is to discretize, with respect to time, the Newton equations of motion. For the MD simulation requirement, the development of a force field describing the interaction potentials between the simulated system particles is of undeniable interest.

### 2.2 Coarse-grained model

**Fig. 1:**
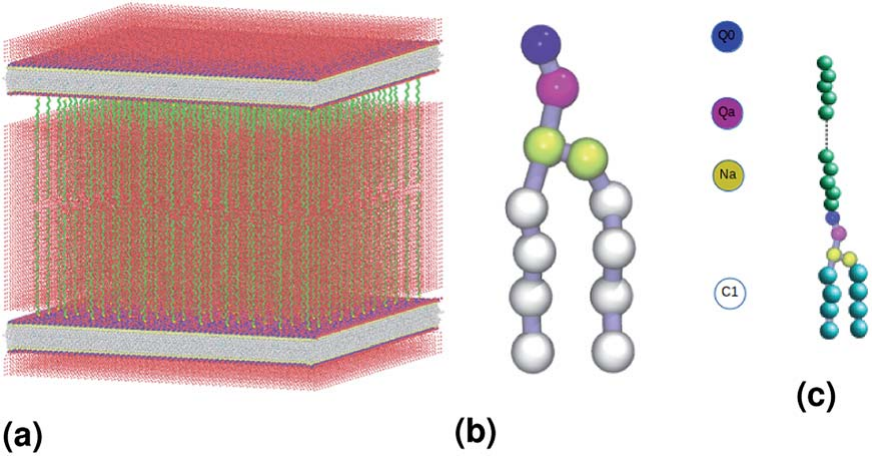
Schematic representation of the studied system, (a) initial distribution of the simulated model used in the molecular dynamics simulation, the lengths of the simulation box are *lx* = 480 Å, *ly* = 480 Å and *lz* = 465 Å. (b) DPPC lipid model. (c) Lipopolymer (DPPC-PEG) model.

We simulate two adjacent DPPC membrane bilayer with PEG grafted polymers in an aqueous environment using the MARTINI force-field. In fact, several coarse-grained models have been proposed to study lipids and polymers. The first GG model used to represent lipid membranes and polymers has been proposed, in 2001, by Klein *et al.*^27,28^ This model was used to study the self-assembly of lipids/peptides, the insertion of hydrophobic nanopores in a lipid bilayer^29^ or the antimicrobial action of a polymer chain.^30^ This set of works combines real statistical mechanics with empirical modeling and included considerable insight. These approaches might not be considered fully rigorous, but they have nevertheless a considerable impact on the results they have produced. Then, Marrink *et al.* proposed another CG model of lipids, which makes it possible to reproduce several lipid phases.^31–33^ This model is an essentially empirical approach to CG modeling. It uses simple functional forms to describe interaction potentials. The parameters of the interaction potentials are determined by adapting to the experimental data, such as the sharing of free energies between oil and water. To simulate membranes and membrane-related phenomena, the MARTINI CG force field appears to be the best choice because the membranes are under the action of amphipathic assembly forces. The MARTINI force field is, therefore, the most suitable field for modeling such systems. Within the framework of this model, the molecules are not represented by individual atoms, but rather by “pseudo-atoms” approaching groups of atoms, such as whole amino acid residues. By decreasing the freedom degrees, much longer simulation times can be studied at the expense of the molecular details. Fig. 1(b) shows the coarse-grain representation of a DPPC-lipid. In this representation, the PC-head-group consists of two hydrophilic groups: choline (blue) and a phosphate-group (pink). The first carries a positive charge (+e), while the second, carries a negative one (−e). The Na particles (yellow) refer to the intermediate polarity glycerol moiety, and the tail double-bonds can be efficiently modeled using slightly less hydrophobic beads, as well as a change in the angle-interaction-potential that governs the rigidity and orientation of the lipid-tails. The MARTINI force field is based on a mapping of one to four, which means that, on average, four heavy atoms, including the associated hydrogens, can be represented by a single coarse-grained bead. Consequently, one coarse-grained water bead corresponds to four water molecules. To properly reproduce the chemical nature of the modeled systems, four main types of the coarse-grained particles are defined, namely, metallic, polar, nonpolar and charged. The four main types of the coarse-grained particles are divided into sub-types, based on hydrogen-bonding capabilities (donor, acceptor, both or none) and polarity (ranging from 1-low polarity to 5-high polarity), giving a total of 18 unique “building blocks”. The described mapping scheme provides a relatively straightforward and effective way of switching from all-atom to coarse-grained representation for a wide range of the biological systems. Interactions between the coarse-grained particles are described by a force field containing terms typical for other classical force fields. In the MARTINI coarse-grained model, each monomer in polyethyleneglycol PEG is represented by a single bead (EG). Fig. 1(c) show schematic representation of a lipopolymer DPPC-PEG, where the DPPC-PEG bond is assumed to be rigid.

### 2.3 Interaction potentials in the MARTINI force field

The proposed model for membranes supported *via* graft polymers consists of lipid bilayers supported by PEG polymer chains, the bilayers are consisting of dipalmitoylphosphatidylcholine lipids (DPPC), a schematic representation is presented in Fig. 1. The simulation used the Martini coarse-grained force field developed by J. Marrink for use with DPPC lipids, water (MW), and PEG polymer.^34,35^

The importance of using the coarse-grained model is to allow the extend of the spatial and temporal scales of the simulations compared to the all-atom model. The MARTINI force field is one of the widely used Coarse-Grained (CG) models in MD simulations. More details about the MARTINI CG force field for lipids and polymers can be found in the literature.^34–40^ Here, we will present the model briefly. In the MARTINI CG model, all particle pairs (in the Martini force field) *i* and *j* at distance *r* interact *via* a shifted Lennard-Jones *V*_LJ_ and coulombic *V*_C_ interactions:2
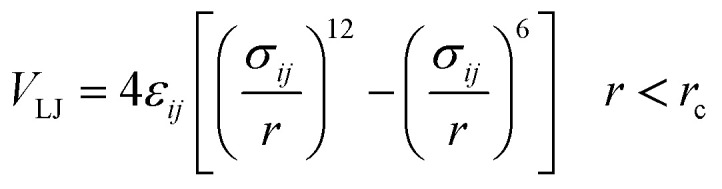
3
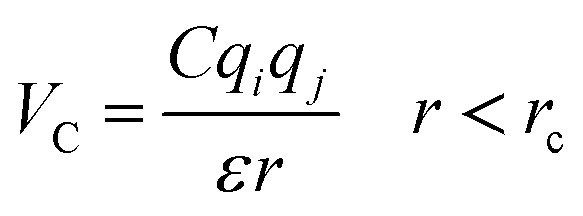


For the DPPC lipids and PEG polymer the bond interactions between adjacent pairs of beads are described by a harmonic potential *V*_b_(*r*):

4 *V*_b_ = *K*_b_(*r* − *r*_0_)^2^

With the interaction parameters between PEG monomers *K*_b(EG–EG)_ = 20.3 kcal mol^−1^ Å^−2^, and *r*_0(EG–EG)_ = 3.3 Å. The bonded interaction parameters for bond DPPC bead are set to *K*_b(*ij*)_ = 24 kcal mol^−1^ Å^−2^ and *r*_0(*ij*)_ = 4.7 Å. The angle interaction between triplets of DPPC beads use a cosine squared harmonic potential *V*_a1_(*θ*)

5 *V*_a1_ = *K*_a1_(cos(*θ*)−cos(*θ*_0_))^2^

With *K*_a1_ = 3 kcal mol^−1^ for all angles, *θ*_0_ = 120° for the *Q*_a_–Na–Na equilibrium angle and *θ*_0_ = 180° for the others angles. The PEG polymer, the a harmonic angle interaction *V*_a2_ is used, and Fourier dihedral interaction potential *V*_d_ is added to represent the PEG chains torsion.6
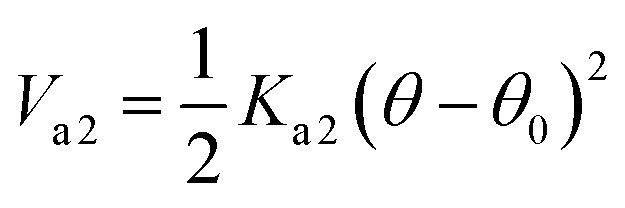


With *K*_a2_ = 20.3 kcal mol^−1^, and *θ*_0_ = 130°.7
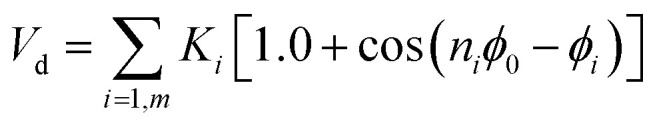


The parameters of the dihedral interaction are given in Table 2.

### 2.4 Simulation details

The principle of MD simulation consists of generating the trajectories of a finite set of particles, by integrating numerically the classical equations of motion. The trajectories thus determined are used to evaluate the static and dynamic properties by temporal averages, which, at very large-times, coincide with the statistical-averages, for ergodic systems. The motion equation of each bead can be integrated numerically using MD simulation, to give the trajectories of each bead in phase space. At each point of the trajectory, any physical quantity, *A*, takes instantaneous value, *A*(*t*). When the system is at thermodynamic equilibrium, *A* becomes stationary, and its average value can be determined within a time interval. Thus, DM makes possible the study of the behavior of the considered system, on average, by calculating its temporal evolution numerically and averaging the quantity of interest over a long enough time. Then, for larger times, physics respects the ergodicity principle stipulating that: 
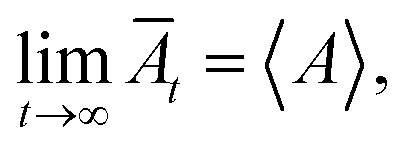
 where *A* designs any physical property and *Ā*_*t*_ represents its temporal mean-expectation-value, and 〈*A*〉 accounts for an equilibrium-average calculated in the adopted statistical ensemble. To study the physical properties of lipid membranes with grafted polymers using the molecular dynamics simulation method based on the MARTINI coarse-grained model, we have simulated a lipid membranes with grafted polymers model consisting of 15 376 DPPC molecules and different PEG lipopolymer molar fraction (0.005, 0.014, and 0.1), fully hydrated by 112 659 water beads. Molecular dynamics simulations were performed using the LAMMPS software package,^41^ In order to eliminate the contributions of surfaces that affect the physical properties of the system, periodic boundary conditions are imposed.^42^ We performed molecular dynamics simulations in the NPHT and NVTE ensembles. *N* is the number of coarse-grained beads, *V* is the volume of the simulated system, *T* is the temperature, *H* is the enthalpy, *E* is the internal energy and *P* is the external pressure. In the first simulation we plan to calculate the equilibrium distance between the two membranes for different molar fractions of lipopolymer at ambient conditions of temperature and pressure. The preformed initial distribution (Fig. 1) is adjusted in local potential energy minimum by iteratively adjusting atom coordinates. Then, the system is equilibrated under the NPHT conditions, by coupling the NPH barostat^43^ to the Langevin thermostat^44^ for one million time-step, with a time-step of 10 fs, we remark that, after a 2 × 10^5^ time-step, the volume remains practically constant and then the polymer layer in the *z*-direction can be calculated. The NPHT simulation updates the position and speed for each time step and changes the volume freely to achieve the imposed equilibrium conditions of external pressure and temperature (*T* = 300 K, *P* = 1 bar). For these conditions, the membrane bilayer is in a gel phase giving an area per lipid *A*_l_ around 0.7 nm^2^. The second simulation is a Brownian dynamics (BD) performed in the NVTE ensemble, it is done by coupling the Langevin thermostat which models an interaction with an implicit background solvent with *T* = 300 K, to the NVE integration scheme which performs a constant NVE simulation to update the position and the speed of atoms,^45^ in this simulation we investigate the structure and dynamics of both DPPC lipids and PEG polymers. The NVTE simulations were run for two million time-step, with a time-step of 10 fs, giving a simulation time of 20 ns.

## 3 Results and discussions

### 3.1 Polymer layer and membrane thickness

Polymer physics theories, starting with Flory^46^ for the free chains and passing to de Gennes^47^ and Alexander,^48^ for grafted polymers, can be used to characterize statistical configurations and the thermodynamics of polymer chains. The study of polymers treated on solid surfaces shows the existence of two regions of concentration of polymers. These are characterized by the so-called “mushroom” and “brush” configurations of the graft polymer chain. The “mushroom” and “brush” regimes are qualified from the visualization of the simulated system and by comparing the polymer layers of the MD simulation with those of the theory.

The mushroom and brush regimes are qualified by qualitatively from the visualization of the simulated system and quantitatively by comparing the polymer layer to that established theoretically and experimentally.

The mushroom regime is due to low concentrations of graft polymer and the brush regime refers to higher concentrations. The reason for using the same parameters to present the PEG used in the experimental work of Evans *et al.*,^49^ in term of the degree of polymerization *n*_p_ = 45 and the monomer radius *a* = 4.3 Å is to validate the MD simulations by comparing its results to the experimental findings. We note that, the investigation of other degrees of polymerization and molar fractions for PEG or for other polymers, polycations or polyanions is can be conducted using the present MD simulations protocol. For a comparison to the theoretical predictions, the configuration of the chain in the two regimes can be treated by the methods of polymer physics, as developed below:

#### 3.1.1 Mushroom regime

**Fig. 2:**
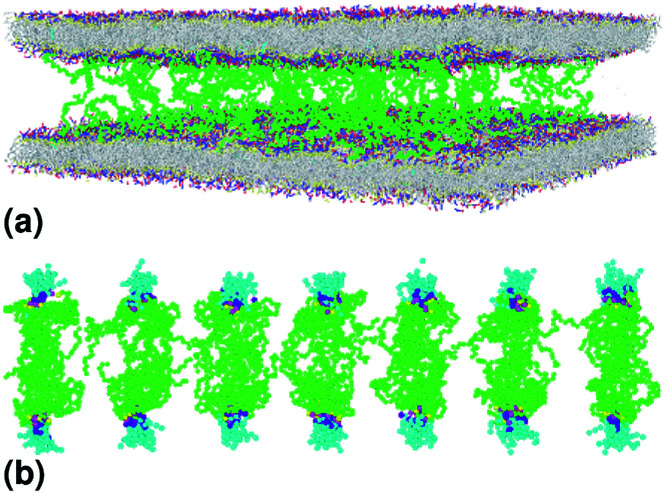
Captions of the NPHT simulation at *T* = 300 K and *P* = 1 atm of the mushroom configuration regime. The model membranes composed of DPPC lipid bilayer are decorated with a molar fractions *X*_p_ = 0.005 of lipopolymer DPPC-PEG with the same degree of polymerization *n*_p_ = 45 and the whole is hydrated by water molecules. After one nanosecond, the simulated system is equilibrated and the membrane–membrane distance remains generally constant (for clarity, the water molecules are not showing). (a) MD Simulation in the mushroom for *X*_p_ = 0.005. (b) Lipopolymer conformation in the mushroom regime.

When the chains are grafted onto a surface with little density, they do not interact and behave almost like isolated grafted chains as shown in Fig. 2a and b. It is customary to call this regime the mushroom regime. As a first approximation, they are not affected by the presence of the surface and their average height is the order of *R*_F_ which is determined by the degree of polymerization *n*_p_ and the size a of the monomer unit. The dimensions of the free polymer are determined by steric interactions (the excluded volume effects) that tend to extend the polymer. These are balanced by the entropic effects unfavorable to the stretching of the coiled polymer chain. In Flory's theory, the free energy of a real chain is given by:8
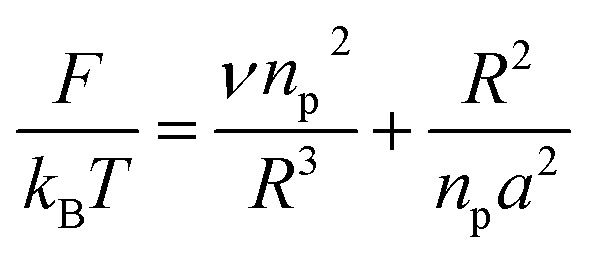
where *R* is the end-to-end distance of the chain and *ν* is the excluded volume by monomer. In general *ν* = *a*^3^(1 − 2*χ*). Where *χ* is the Flory–Huggins interaction parameter with an athermic solvent, for which non-steric intramolecular interactions may be neglected *ν* ≈ *a*^3^. The first term of eqn (8) represents the excluded volume interactions, which are represented in the mean field approximation and are proportional to the square of the local concentration. The second term of the eqn (8) represents the free energy of stretching, which is derived from the entropy of an ideal polymer chain whose extremities undergo a random (Gaussian) walk.^48^ Free energy in eqn (8) with respect to *R*, gives the following expression for the Flory radius (three-dimensional):9
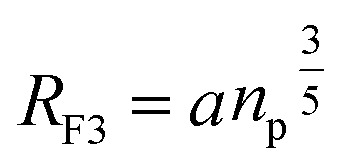


Hristova and Needham^50^ find that for the PEG monomer unit of oxyethylene: *a* ≈ 0.35 nm. From the adhesion measurements, Evans *et al.*^49^ found that the data for PEG lipids is better adjusted by a value of *a* ≈ 0.43 nm. In this work, for a reason to compare the coarse-grained MD simulation results with the experimental findings, we simulate PEG polymers with a degree of polymerization *n*_p_ = 45 and different molar fractions, similar to the works of Evans *et al.*^49^

#### 3.1.2 Transition from the mushroom regime to the brush regime

The transition between mushroom and brush regimes occurs at the graft lipid concentration for which the polymer chains associated with the surface begin to overlap. This condition is satisfied approximately to polymer molar fractions 
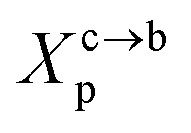
 such as:10
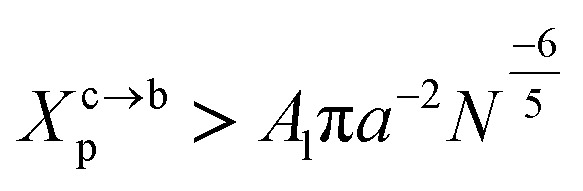
where *A*_l_ is the area of the membrane by lipid (*A*_l_ = 0.6–0.7 nm^2^) for lipid in the fluid phase and (*A*_l_ = 0.432 − 0.48 nm^2^) in the gel phase. For a graft polymer with molecular weights of 2000, *i.e.* (*n*_p_ = 45) for the PEG polymer, the transition between the mushroom and brush configurations must take place at 
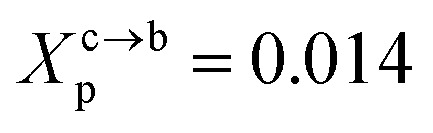
 for fluid phase membranes.

#### 3.1.3 Brush regime

**Fig. 3:**
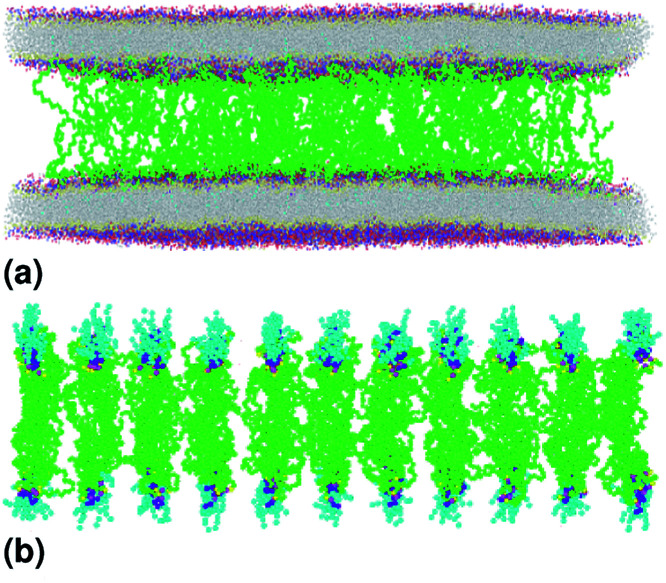
Captions of the NPHT simulation at *T* = 300 K and *P* = 1 atm of the critic configuration regime. The model membranes composed of DPPC lipid bilayer are decorated with a molar fractions *X*_p_ = 0.014 of lipopolymer DPPC-PEG with the same degree of polymerization *n*_p_ = 45. (a) MD Simulation in the critic regime for *X*_p_ = 0.014. (b) Lipopolymer conformation in the critic regime.

**Fig. 4:**
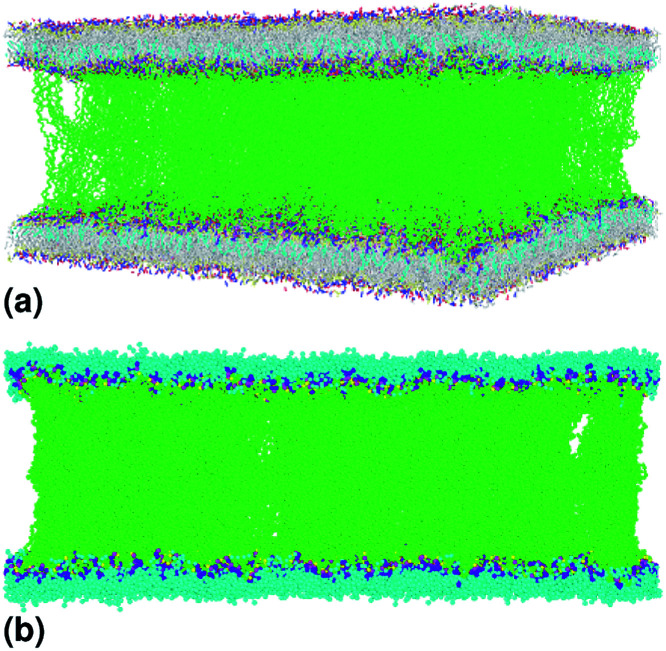
Captions of the NPHT simulation at *T* = 300 K and *P* = 1 atm of brush mushroom configuration regime. The model membranes composed of DPPC lipid bilayer are decorated with a molar fractions *X*_p_ = 0.1 of lipopolymer DPPC-PEG with the same degree of polymerization *n*_p_ = 45 and the whole is hydrated by water molecules. After one nanosecond, the simulated system is equilibrated and the membrane–membrane distance remains generally constant (for clarity, the water molecules are not showing). (a) MD Simulation in the brush regime for *X*_p_ = 0.1. (b) Lipopolymer conformation in the brush regime.

A polymer brush consists of polymer chains, one and only one end of which is covalently bonded to a solid substrate, as schematically shown in Fig. 4a and b. As the concentration of the graft polymer increases, the polymer head groups begin to interact and present a more stretched configuration (like a brush) in which the polymer chains extend from the surface of the membrane (Fig. 3 and 4).

##### 3.1.3.1 Mean field theory for the brush regime

Two different treatments have been adopted to study the polymer chain bond in the brush regime. The first is based on the average field theory which, in essence, is similar to that presented in eqn (8). The second is the theory of scale laws that has been proposed by de Gennes. The average field theory is presented in this section and the theory of scaling laws in the next section. In the brush regime, the polymer chains are extended and the problem is essentially unidimensional. The chains are confined in the normal direction to the grafting surface (Fig. 2a and b). For a grafted polymer chain, the one-dimensional version of the average field expression for free energy is:^51^11
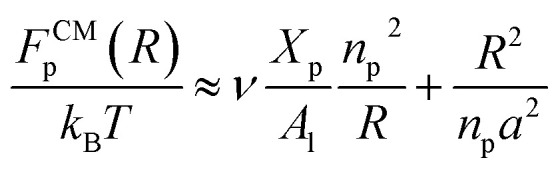
where *X*_p_ is the molar fraction of the lipid polymer and *A*_l_ is the area per lipid. Minimizing the free energy of eqn (11) with respect to *R* (end-to-end distance of the chain) gives the following expression for the equilibrium length of the polymer brush:12
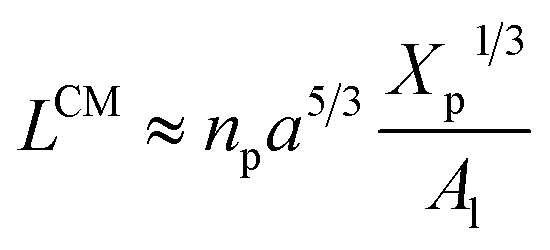


##### 3.1.3.2 Scale theory for the brush regime

The essential determinant in scale law theory is the one-dimensional nature of the problem for confined polymer chains in the brush regime (Fig. 2a and b). Under these conditions, the length *L* of the polymer chains is linear in degree of polymerization *n*_p_. For long polymer chains, the scale law for the equilibrium length is given by:^47,52^13
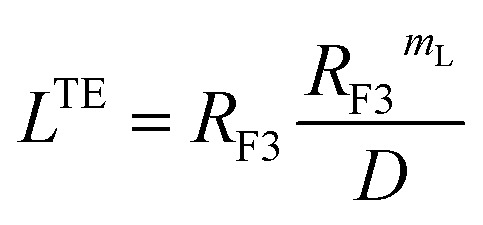
where *D* is the diameter of the confinement, *i.e.* the distance between the grafting points of the polymer. The scale of length in the problem is the Flory radius *R*_F3_, given by eqn (6). The requirement that *L*^TE^ be linear in *n*_p_ gives for the exponent the value *m*_L_ = 2/3. In terms of the molar fraction of the polymer lipid, the distance *D* between the grafting points is given by 
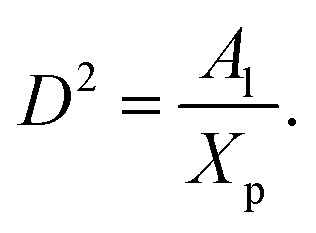
Eqn (10) then becomes:14
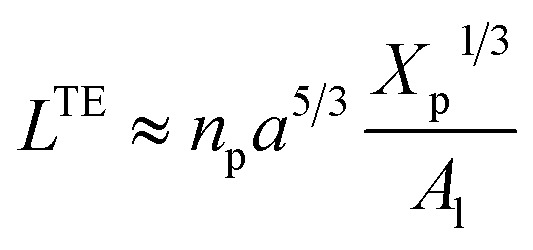


**Fig. 5:**
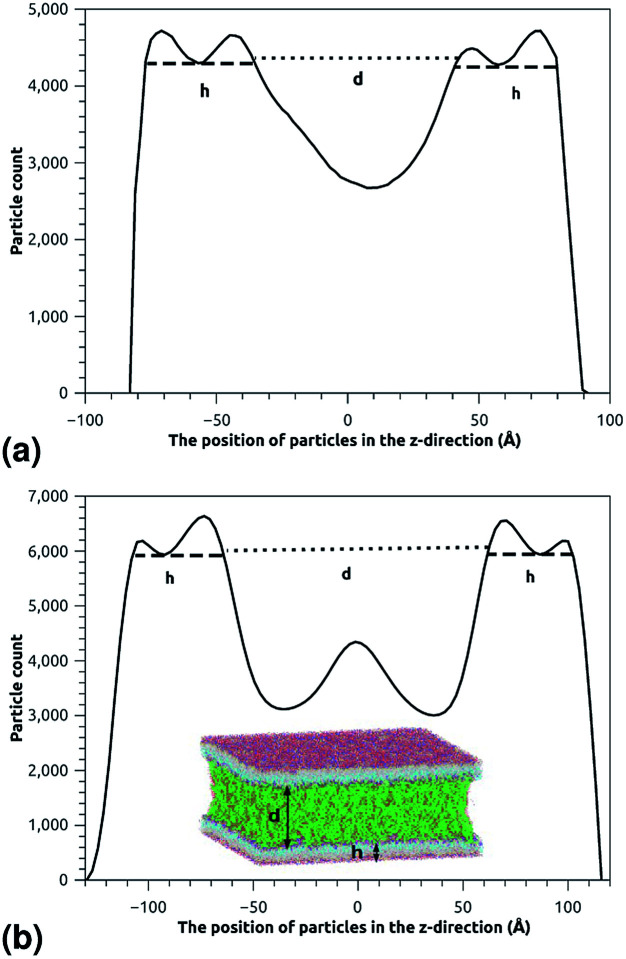
The particle-count in the *z*-direction of the simulated system for the two essential regimes of polymers configuration, where *h* is the membrane thickness and *d* the distance between the two adhesive membranes. The polymer thickness is calculated as *L* = *d*/2. (a) The particle-count in the *z*-direction in the mushroom regime. (b) The particle-count in the *z*-direction in the brush regime.

This is exactly the same result as obtained from the mean field theory given in eqn (9). In Table 1 the thickness of the polymer layers available experimentally, for the PEG polymer with a degree of polymerization *n*_p_ = 45 and different molar fractions, by A. K. Kenworthy *et al.*^53^ and DPPC bilayer thickness measured by N. Kucerka *et al.*^54^ is compared to that obtained by the present MD simulation at the NPHT statistical ensemble under the ambient condition of pressure and temperature, for different molar fractions of lipopolymer (0.005, 0.014 and 0.1) using a lipopolymer composed of a DPPC molecule bound to a PEG polymer chain of degree of polymerization *n*_p_ = 45 and monomer radius *a* = 0.43 nm, for this parameters the transition mushroom-brush lipopolymer molar fraction is *X*_p_ = 0.014, from Fig. 2, it is clear that for *X*_p_ = 0.005 the lipopolymer shows a mushroom regime, for *X*_p_ = 0.014 shows a critical regime and for *X*_p_ = 0.1 shows a brush regime. From molecular dynamics simulation, as the membranes are more congested for comparison to the diluted polymers, we can deduce the polymer and membrane thickness by analyzing the density profile of the simulated system using the Ovito visualization tool.^55^ We find that the thickness of the polymer increases with the increase of the molar fraction of the graft polymers and that the thickness of the membrane decreases a little from the mushroom regime to the brush regime. Thus, the polymer layer, which covers the liposomes, plays an important role in its protection. The incorporation of lipopolymers prevent the adhesion liposome–liposome even with a small molar fraction of grafted polymers, and they form a layer which protects the liposome from supposed system immune attack (Fig. 5 and Table 3).

**Table 1:** Non-bonded interaction potential parameters

Pair type	*σ* (Å)	*ε* (kcal mol^−1^)
*Q*_0_–*Q*_0_, *C*_1_–*C*_1_	4.7	0.836521
*Q*_a_–*Q*_a_, *Q*_a_–EG, MW–MW	4.7	1.195030
Na–Na, *Q*_0_–Na, *Q*_a_–Na, *Q*_0_–EG	4.7	0.956024
EG–EG	4.3	0.806644
*Q*_0_–*Q*_a_	4.7	1.075527
*Q*_0_–*C*_1_, *Q*_a_–*C*_1_	6.2	0.478012
MW–*C*_1_	4.7	0.478012
Na–*C*_1_, EG–*C*_1_	4.7	0.645316
*Q*_0_–MW, *Q*_a_–MW	4.7	1.338434
Na–MW	4.7	0.956024
EG–Na	4.7	1.075527
EG–MW	4.7	1.075527

**Table 2:** Dihedral interaction potential parameters

*ϕ*_0_(°)	*K*_*i*_ (kcal mol^−1^)	*n*_*i*_
180	0.46845	1
0	0.046845	2
0	0.078872	3
0	0.028681	4

**Table 3:** Thickness of the polymer layer obtained by MD simulations for DPPC-PEG_*n*_p__ lipopolymer in DPPC bilayer membranes

*X*_p_	Regime	*L*_Exp_ (nm)	*L*_DM_ (nm)	*h*_DM_ (nm)
0.005	Mushroom	3.75	3.6 ± 0.2	4.1 ± 0.05
0.014	Critic	—	4.3 ± 0.2	4.05 ± 0.05
0.1	Brush	6.6	6.4 ± 0.2	4.0 ± 0.05

### 3.2 Radial distribution function and mean-field interaction potential

**Fig. 6:**
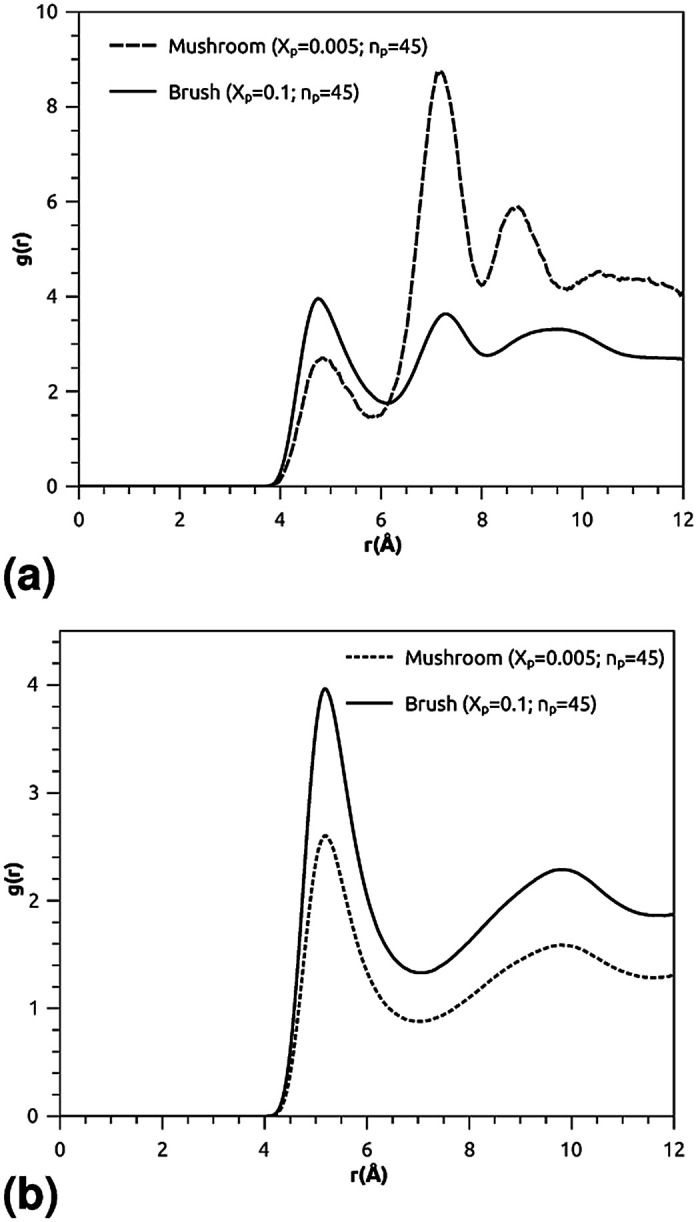
Radial distribution function (RDF): (a) RDF of the group of lipopolymer molecules, (b) RDF of the group of lipid molecules.

**Fig. 7:**
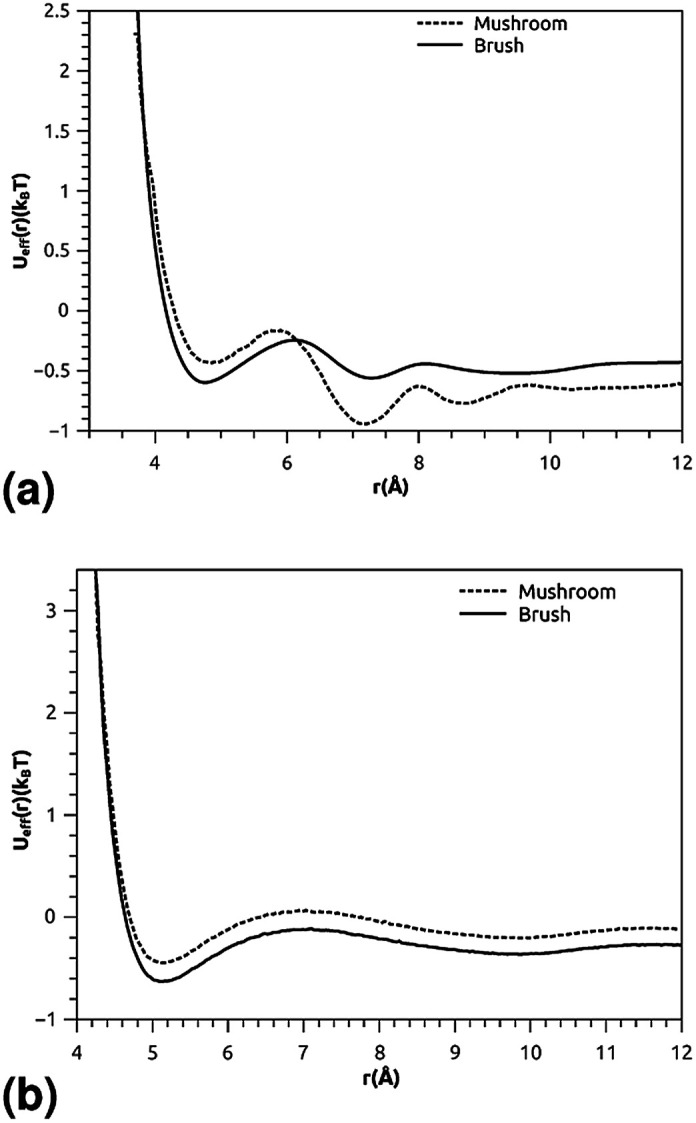
The mean-field interaction potential (*U*_eff_(*r*)), (a) *U*_eff_(*r*) of the group of lipopolymer molecules, (b) *U*_eff_(*r*) of the group of lipid molecules.

The investigation of the three-dimensional distribution of the beads of DPPC lipids and PEG polymer, for the two grafting regimes, is carried out by analyzing the radial-distribution-function (RDF) from MD simulations in the NVTE statistical ensemble. RDF can be defined as the probability of finding a coarse-grained bead distributed around a given (central) bead, in three dimensions. In fact, RDF provides information on the local distribution of the coarse-grained beads in a group among the various beads of the system. Analytically, RDF is defined by the following thermal-average:15
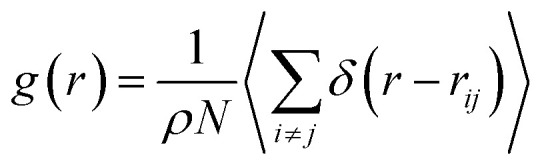
where *ρ* is the number-particle-density. With *ρ* = *N*/*V*, where *N* is the number of constituents (DPPC-molecules or PEG polymer beads) and *V* is the volume of the simulation box. The above sum is performed over all pairs of constituents. Fig. 6a and b shows the radial distribution function (RDF), which gives the probability of finding a coarse-grained bead at a distance *r* from another bead of the same considered group of molecules (lipids or polymers). Inspection of this radial distribution function confirms that, for brush and mushroom regimes, the membrane bilayer is in the gel phase, the main peaks of the RDF between lipids increase proportionally to the graft polymer molar fraction, *i.e.* the membrane lipids are more congested for the brush regime, for the reason that the interaction between lipids is more attractive in the brush regime than the mushroom regime. For polymers, in the brush regime, the polymer RDF has narrow peaks corresponding to an ordered monomers distribution, and in the mushroom regime, the polymer RDF has large peaks, which means that it has a messy structure. Fig. 7a and b shows the effective mean-field interaction potential computed as *U*_eff_/*k*_B_*T* = −log(*g*(*r*)) for polymers and lipids in the brush and mushroom regimes. In the mushroom regime, the interaction between the monomers is an attractive type. However, when the concentration of the grafted polymer increases from the mushroom regime to the brush regime, the interaction between the monomers becomes less attractive and the polymer head groups present a stretched configuration. The interaction between lipids is more attractive in the brush regime. Thus, the stability of DPPC lipids bilayer increases with the increase of lipopolymer molar fraction.

### 3.3 Dynamic aspect

#### 3.3.1 Lipids and polymer dynamic

Membrane bilayer systems containing lipopolymers are idealized model systems designed to study fundamental physical properties of liposome. From an experimental point of view, these systems can indeed be synthesized and analyzed by different tools, such as neutron scattering or fluorescence correlation and pulsed-gradient spin-echo nuclear magnetic resonance. The MD simulation is capable of determining the type of diffusion in which the model membranes are decorated with a different molar fraction of membrane lipopolymers.

##### 3.3.1.1 Lipids dynamic

The lipids dynamic can be described by analyzing the mean square displacement defined as:16
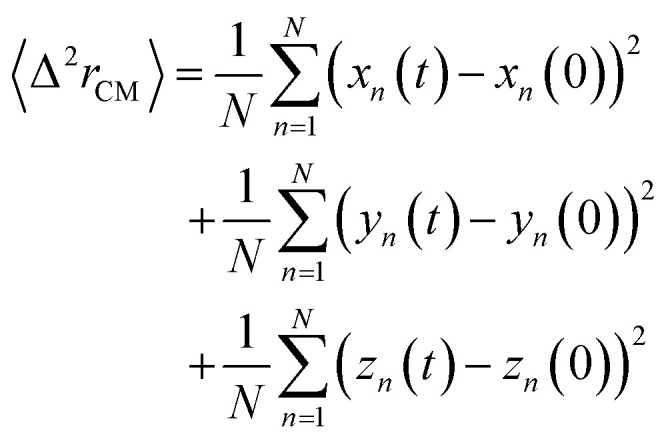


The 〈Δ^2^*r*_CM_〉 is found proportional to the diffusion coefficient *D* and the diffusion exponent *α* for a system of dimension *d* by:^56^

17 〈Δ^2^*r*_CM_〉 = 2*dD*_*α*_*t*^*α*^

which can be expressed as:

18 log(〈Δ^2^*r*_CM_〉) = log(2*dD*_*α*_) + *α* × log(*t*)

where the log function refers to the common logarithm. From the log–log plot of MSD *versus* time we determine the diffusion coefficients *D*_*α*_ and the exponent *α* < 1 related to the different diffusion regimes. We underline that subdiffusion is a feature of overcrowded systems, where the trajectories of their mobile constituents are strongly correlated. Note that the scale relationship above is valid for significant times, that is, beyond a characteristic time depending on the specific details of the broadcast process and the host support structure. This long-term behavior deviates from the linear dependence on time found for Brownian motion.^57^ Here, *D*_*α*_ represents the generalized diffusion coefficient or (the fractional diffusion coefficient). The latter is expressed in length^2^/time^*α*^ unit. We underline that subdiffusion is a characteristic of saturated systems, where the trajectories of their mobile constituents are strongly correlated. Note that the scale relationship above is valid for significant times, that is, beyond a characteristic time depending on the specific details of the broadcast process and the host support structure. In general, a particle is said to be subdiffusive if the condition 〈Δ^2^*r*_CM_(*t*)〉(*t*)/*t*→0, for *t* → +∞, is satisfied (very slow diffusion). This explains why the exponent *α* must be in the range 0 < *α* < 1. From an experimental point of view, subdiffusion has been observed in many scientific fields.^57^ Subdiffusion originates from the cage effect,^57^ where the tracer is trapped in a cage formed by its neighbors. It has been found that the length of stay, Δ*t*_c_, of a tracer in the cage, composed of *N* neighbors, is noted: Δ*t*_c_ ∼ *N*^1/*α*^, where *α* is the abnormal exponent.

##### 3.3.1.2 Polymer dynamic

In the coarse-grained representation, PEG chains can be understood as a sequence of *N* beads, EG-monomers, joined by a stretching potential.^58^ The dynamics of the PEG is modeled by the Brownian motion of EG-monomers. The polymer dynamics model, in the coarse-grained scale, was first proposed by Rouse. In the framework of the Rouse model, the equation of motion of the EG-beads is described by the Langevin equations for segmental motion:19
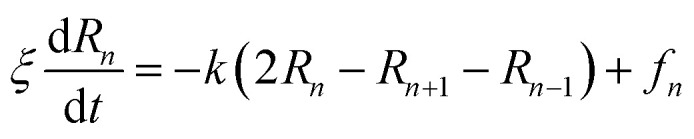
where, (*R*_1_, *R*_2_,…, *R*_*N*_ ≡ *R*_*n*_) is the position of the beads, *k* = 3*k*_B_*T*/*a*^2^ is the entropic spring force and *f*_*n*_ a random force. We assume that each bead feels the same friction *ξ*, that its motion is overdamped, and that the diffusion coefficient *D* = *k*_B_*T*/*ξ* is independent of the position *R*_*n*_ of the bead.

The random force *f*_*n*_ verifies the conditions:^59^

20 〈*f*_*n*_(*t*)〉 = 0

21 〈*f*_*n*_(*t*)*f*_*m*_(*t*′)〉 = 6*ξk*_B_*Tδ*(*t*−*t*)*δ*_*nm*_

The solution of the Langevin equation gives the following behavior of the mean-square-displacement as a function of time.22
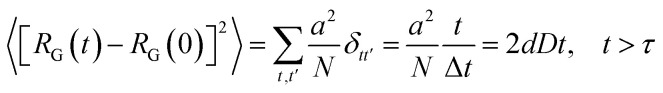
with *τ* is a characteristic time for wich the motion of the center of mass fallow a normal diffusion law, in a space of dimension *d*, with a diffusion constant *D*.

On the other hand, the polymers can show a subdiffusion due to the cage effect. For this reason, the diffusion of the center-of-mass is not normal. In this case, the mean-square-displacement of a monomer is given by:

23 〈Δ*r*_CM_^2^〉 = 〈[*R*_G_(*t*) − *R*_G_(0)]^2^〉 ≃ 〈[*R*_*n*_(*t*) − *R*_*n*_(0)]^2^〉 ∝ √*t*,** ***t* < *τ*

#### 3.3.2 Analysis of MD results

**Fig. 8:**
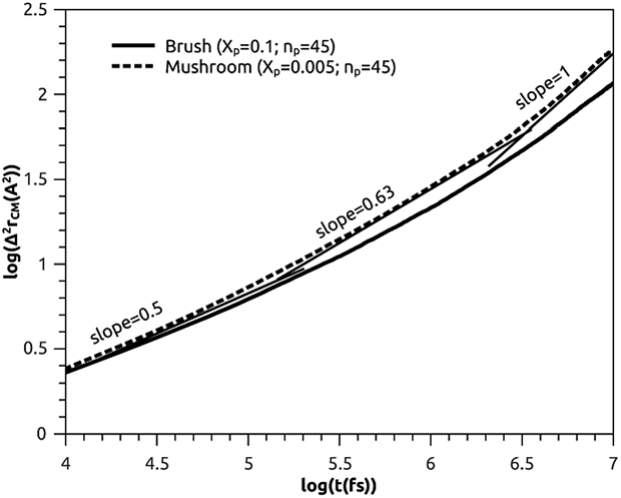
Mean square displacement (MSD): log–log of MSD as a function of time for DPPC molecules.

**Fig. 9:**
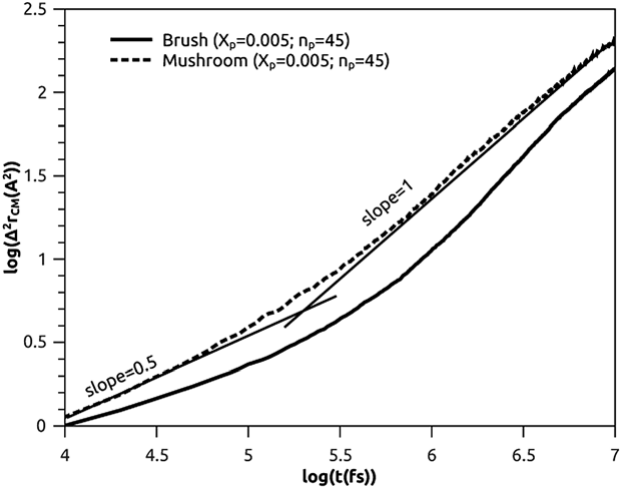
Mean square displacement (MSD): log–log of MSD as a function of time for lipopolymers molecules.

To study the influence of the molar fraction of the grafted polymer on their dynamics, and then on the membrane dynamics, we keep their degree of polymerization fixed to the values: *n*_p_ = 45, and vary the molar fraction. Fig. 8 and 9 show the results of the molecular dynamics simulations in the NVTE statistic ensemble, for both brush and mushroom regimes, the temperature is fixed at 300 K. The diffusion coefficients *D*_*α*_, that are determined by extrapolating the log–log plot of the MSD as a function of time, are given in Table 4. In Fig. 8, we depict the log–log plot of MSD against time for polymers. We first remark that after a short initial regime of subdiffusive motion (*t* = 100 ps), with a diffusion exponent *α* = 0.5, the polymer dynamics reach the normal diffusion, after this time MSDs are straight lines of the same slope *α* = 1. The PEG dynamics, for the mushroom regime, is in perfect agreement with the analytical prediction in the framework of the Rouse model. However, for the brush regime, PEG dynamics, at short time, show a deviation from the Rouse model. In this regime, the polymer density is high, and can cause a cage effect that blocks the monomers movement and therefore, the exponent 
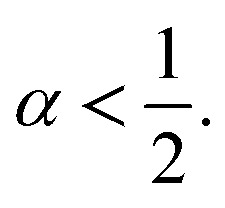
 Experimental values of the diffusion coefficient of the PEG polymer were found by Waggoner *et al.*^60^ For a low molecular weight, the diffusion coefficient is of the order of 10^−6^ cm^2^ s^−1^ and for a large molecular weight, it is of the order of 10^−7^ cm^2^ s^−1^. Our coarse-grained simulation for branched PEGs is in good agreement with the experimental findings. In Fig. 9 we depict the log–log plot of MSD against time for lipids, It is well known that the membrane bilayer at ambient temperature appears to be in a gel phase characterized by a slow lateral diffusion of lipids, the diffusion of lipids is anomalous. This is demonstrated in MSD curves depicting the time-dependent anomalous diffusion exponent (*α* < 1), *α* remains <1 even for long time. We remark a slight decrease of the membranes dynamics passing from the mushroom to the brush regime. The values obtained experimentally of the diffusion coefficient in the plane of a gel membrane are of the order of 10^−7^ cm^2^ s^−1^,^61^ MD results show that the polymer coating to the membranes has no important influence on the lipids dynamics properties. The dynamics behavior of lipids can be described, as well as polymers, by the Rouse model. But, the normal diffusion expected by Rouse appears at very long time (*t* ≳ 10) ns, as observed by Flenner and coworkers in an all-atom MD simulation of the DMPC bilayer membrane.^62^ In this work, we are interested in the subdiffusion phenomenon. The simulation time is of 20 ns and it is sufficient to explore the anomalous dynamics, which takes place for a short time *t* < 10 ns. At long simulation time, this regime disappears and the normal diffusion takes place. We recall that lipids show a long time subdiffusion. In contrast, the polymers have a normal diffusion after a short regime of subdiffusion, even in the brush regime, where the molar fraction of coating polymers is important. Furthermore, it should be noted that, for a macroscale viewpoint where the liposomes can be viewed as dispersed colloidal microspheres, the polymer plays an important role in preventing the liposomes aggregation and in facilitating their transport (Table 5).^63^

**Table 4:** Diffusion coefficient *D*_*α*_ of DPPC lipids obtained by MD simulations in the NVTE statistical ensemble

Regime	*t* (fs)	*α*	(*D*_*α*_) (cm^2^ s^−*α*^)
Mushroom	[0, 10^5.3^]	0.50	7.4 × 10^−4^
	[10^5.3^, 10^6.4^]	0.63	1.2 × 10^−4^
	[10^6.4^, 10^7^]	1.00	1.4 × 10^−6^
Brush	[0, 10^5.6^]	0.42	6.9 × 10^−4^
	[10^5.6^, 10^7^]	0.63	9.6 × 10^−5^
	[10^6.6^, 10^7^]	1.00	8.9 × 10^−7^

**Table 5:** Diffusion coefficient *D*_*α*_ of polymer obtained by MD simulations in the NVTE statistical ensemble

Regime	*t* (fs)	*α*	(*D*_*α*_) (cm^2^ s^−*α*^)
Mushroom	[0, 10^5.2^]	0.50	2.8 × 10^−4^
	[10^5.2^, 10^7^]	1	1.4 × 10^−6^
Brush	[0, 10^5.4^]	0.34	2.2 × 10^−4^
	[10^5.4^, 10^7^]	1	8.5 × 10^−7^

## 4 Conclusion

We studied the polymer layer, the structural and dynamic properties of a liposome model designed for drug delivery using coarse-grained molecular dynamics simulations at two different statistical ensembles. From the NPTH MD simulations, it has been observed that the distance of the polymer layer increases with the increase of the molar fraction of the graft polymers and shows a transition from the mushroom regime to the brush regime. When polymer chains are grafted onto the surface of the membrane with a small molar fraction, they do not interact and behave almost like isolated chains. It is customary to call this regime the mushroom regime. As a first approximation, the graft polymers are not affected by the presence of the surfaces, for ambient conditions of pressure and temperature, and their average height is of the order of *R*_F_ which is determined by the degree of polymerization *n*_p_ and the size of the monomer unit. As the concentration of the graft polymer increases, the polymer beads of different chains interact and exhibit a more stretched configuration (such as a brush) in which the polymer chains extend from the surface of the membrane. The results obtained using the MD simulation are in good agreement with the experimental findings from X-ray diffraction. From the NVTE MD simulations, the analysis of the radial distribution function has shown that the modification of the molar fraction of lipid graft polymers changes the interaction of the hydrophobic chain. For the brush regime, the interaction of the hydrophobic chain is more attractive within the bilayer, resulting in a more close-packed and rigid membrane. The analysis of the mean-square-displacement *versus* time revealed that the dynamics of the membrane slowing during the transition from the mushroom regime to the brush regime. The polymers move freely following a normal diffusion and then, from a macroscopic scale viewpoint, increase the dynamic flexibility of the liposomes by preventing the macroscopic aggregation of liposomes. The diffusion coefficients calculated using NVTE MD simulation are of the order of those measured experimentally using pulsed-gradient spin-echo nuclear magnetic resonance method and single-particle tracking experiments for PEG polymers and DPPC lipids, respectively. We recall that the present nanoscale results can be extended to the industrial scale and proposing techniques of encapsulation of fragile pharmaceutical compounds in nanocapsules. Because of their properties, liposomes covered with biocompatible polymers are ideal for protecting and transporting sensitive pharmaceutical compounds such as those used in cancer therapy.

## Conflicts of interest

There are no conflicts to declare.

## Supplementary Material
